# Breastfeeding reduces the risk of breast cancer: A call for action in high‐income countries with low rates of breastfeeding

**DOI:** 10.1002/cam4.5288

**Published:** 2022-09-26

**Authors:** Britta Stordal

**Affiliations:** ^1^ Department of Natural Sciences Middlesex University London London UK

**Keywords:** BRCA1/2 mutation, breast cancer, breastfeeding, pregnancy, risk, triple‐negative breast cancer

## Abstract

Women in the UK have a 15% lifetime risk of developing breast cancer. Like other high‐income countries, women in the UK are having children later in life which increases their risk. The risk of breast cancer is reduced by 4.3% for every 12 months of breastfeeding, this is in addition to the 7.0% decrease in risk observed for each birth. Breastfeeding reduces the risk of Triple‐Negative Breast Cancer (20%) and in carriers of BRCA1 mutations (22–55%). The mechanisms of reduced risk as a result of pregnancy are related to changes in RNA processing and cellular differentiation. The UK has a low rate of breastfeeding (81%) and this is contrasted to countries with higher (Sweden, Australia) and lower rates (Ireland). The low UK rate is in part due to a lack of experience in the population, todays grandmothers have less experience with breastfeeding (62%) than their daughters. An estimated 4.7% of breast cancer cases in the UK are caused by not breastfeeding. The UK only has 43% of maternity services with full Baby‐Friendly accreditation which promotes compliance with the WHO ‘Ten Steps to Successful Breast Feeding’. Legislation in the UK and Europe is far short of the WHO Guidance on restricting the advertising of formula milk. Expansion of the Baby‐Friendly Hospital Initiative, stricter laws on the advertising of formula milk and legislation to support nursing mothers in the workplace have the potential to increase breastfeeding in the UK. Women with a family history of breast cancer should particularly be supported to breastfeed as a way of reducing their risk.

## 1 INTRODUCTION

Breastfeeding has enormous benefits for both the mother and child. Babies who are breastfed have a reduced risk of death from infectious diseases,^1^ hospitalisation for diarrhoea,^2^ and fewer respiratory^2^ and ear infections.^3^ Children who were breastfed may have a reduced risk of asthma and allergic rhinitis but the evidence for this is not as strong.^4^ Children and adults who were breastfed have a reduced risk of obesity and type 2 diabetes.^5^ Mothers who breastfeed have a reduced long‐term risk of cardiovascular disease,^6^, ^7^ diabetes,^8^, ^9^ breast^10^, ^11^ and ovarian cancer.^10^ The longer the duration of breastfeeding the greater the reduction in risk of disease.^7^, ^8^, ^9^, ^10^

## 2 WOMEN IN THE UK HAVE RELATIVELY SMALL FAMILIES STARTING AT AN OLDER AGE

The average age for a woman's first birth is similar across the UK, 28.8 (England and Wales),^12^ 28.7 (Scotland)^13^ and 29.2 (Northern Ireland).^14^ The average age of all women giving birth is also similar across the UK, 30.7 (England and Wales),^15^ 30.9 (Scotland)^16^ and 31.1 in (Northern Ireland).^14^ The average age of birth has been steadily increasing since the 1970s and the UK experience is similar to other high‐income countries.^17^ Two child families remain the most common family size in England and Wales (37%).^18^ Whether through choice or circumstance 20% of women do not have children by age 44 in the UK.^19^ Overall, Europe has seen an increase in women who do not have children, but the rate in the UK is higher than many countries.^19^

## 3 THE UK HAS A LOW RATE OF BREASTFEEDING

Defining the rate of breastfeeding is complex. It is straightforward to understand exclusive breastfeeding or no breastfeeding but there are many versions of combination feeding in between these two extremes. The category of ‘ever breastfed’ is used to allow comparisons globally.^20^ This category is diverse as it will include babies who were breastfed for a few days as well as those breastfed for a year. Breastfeeding data is also influenced by how it is collected, some is routinely collected on every baby born,^21^ whereas others are dependent on mothers responding to a survey and may not reflect the population as a whole.^22^

In the UK women have low rates of breastfeeding, 81% of babies are ever‐breastfed.^20^ Higher rates of ever breastfeeding occur in other high‐income countries such as Australia (92%) and Sweden (98%).^20^ Although the rate of ever breastfeeding is similar in the USA (79%) and much lower in Ireland (55%).^20^

In England, 72.7% of babies born at term had breastmilk as their first feed.^21^ This is similar to the UK ever‐breastfed rate.^20^ In Scotland 75% of mothers reported ever breastfeeding their babies.^22^ Although, the true figure may be much lower as data collected by Health Visitors showed only 66% of Scottish babies were ever breastfed.^23^ In Northern Ireland breastfeeding is attempted for 60% of babies while in hospital and only 46% are breastfed on discharge.^24^ Across the UK breastfeeding is less common in younger mothers and in more deprived areas.^23^, ^24^, ^25^, ^26^

## 4 PREGNANCY AND BREAST‐CANCER RISK

Women in the UK born after 1960 have a 15% lifetime risk of developing breast cancer.^27^ Postmenopausal women with one child have a 13% reduced risk of breast cancer compared to women without children.^28^ Women with two and three children had a 19% and 29% reduced risk respectively.^28^

However, the protective effects of pregnancy is dependent on the age a woman's first birth occurred. Young age of first full‐term pregnancy (<25) reduces the risk of breast cancer in postmenopausal women by 35% compared to nulliparous women.^29^ When a first birth occurred at age 25–29, women had an 11% increased risk compared to women who gave birth at <20 years.^28^ When a first birth occurs at over 30 years there is a 24% increase in breast‐cancer risk.^28^ This is of concern as the age of first birth is rising in high‐income countries. In the UK 19.4% of first births are to women over the age of 29.^30^

While this increased breast‐cancer risk for older mothers is concerning, what we are actually seeing is a reversal of the protective effects of pregnancy for young women. Women who have one child between the ages of 30 and 34 have the same risk nulliparous women.^31^, ^32^ Women who have one child over the age of 35 have a slightly increased risk.^31^ The real increase in risk is seen in women who have multiple full‐term pregnancies aged over 35 years; there is a 57% increased risk compared to women with only one child.^31^

Pregnancy is protective against breast cancer in the long‐term, but in the short‐term both the incidence of breast cancer and the aggressiveness of cancers that do occur increases.^33^, ^34^ The increased risk peaks at about 5 years after birth, but remains elevated for around 20 years.^34^ Women without a family history of breast cancer experience increased risk with births over the age of 30.^35^ Women with a family history of breast cancer experience increased risk regardless of their age giving birth.^35^

Given the reduction in breast cancer risk when a woman's first pregnancy occurs at less than 30 years it would be prudent to include information on breast cancer risk with fertility education in schools.^36^ However, there are complex social reasons why the age of first birth has been steadily increasing in high‐income countries.^19^ Research on women who have turned to IVF due to infertility, in part as a result of delayed childbearing, found that 46% would not have changed their childbearing plans even with better fertility education.^37^

## 5 BREASTFEEDING AND BREAST‐CANCER RISK

A large meta‐analysis of 47 studies from 30 countries examined the impact of breastfeeding on breast‐cancer risk.^38^ The relative risk of breast cancer decreased by 4.3% for every 12 months of breastfeeding, which was in addition to the 7.0% decrease in risk observed for each birth.^38^ The decreased risk of breast cancer associated with breastfeeding was the same in high and low income countries and did not vary with age, menopausal status, ethnic group or age at first birth.^38^ The data truly indicate that breastfeeding universally decreases breast‐cancer risk.^38^ In the context of a high‐income country such as the UK, a woman who has 2 children and breastfed for 12 months with each child will have reduced her risk of breast cancer by 8.6%.

Breastfeeding has been shown to not alter the protective effect of multiple pregnancies in women who had their first full‐term pregnancy before 25 years.^29^ In contrast, women who had 3 or more children, with their first full‐term pregnancy after 25 years had a 106% increased breast‐cancer risk if they did not breast feed.^29^ An estimated 4.7% of breast cancer cases in the UK are caused by not breastfeeding.^39^ The percentages are higher in Scotland and Northern Ireland; 5.2% and 5.9% respectively^39^ which corresponds with lower rates of breastfeeding described earlier.^23^, ^24^ In Australia, an estimated 1.7% of breast cancer cases are caused by lack of breastfeeding, this corresponds with the higher breastfeeding rate in Australia.^40^

## 6 HOW DOES PREGNANCY AND BREASTFEEDING REDUCE THE RISK OF BREAST CANCER?

The protective effects of an early full‐term pregnancy and breastfeeding have been consistently seen in multiple countries and ethnic groups, suggesting that the protection results from biological changes in the breast rather than environmental or socioeconomic factors.^38^, ^41^

The human breast goes through remarkable changes during a woman's lifetime. During puberty increased levels of oestrogen and progesterone causes the breasts to enlarge. This is due to the development of the mammary glands and increased fatty tissue.^42^ The breast contains 15–20 units called lobes, the lobes of the breast drain into lactiferous ducts which lead to the nipple.^43^ Each lobe of the breast is made up of 20–40 lobules, and each lobule consists of 10–100 hollow cavities called alveoli. The alveoli are lined with epithelium which synthesises the protein and lipid components of breast milk.^43^ Elevated hormone levels during pregnancy causes the ductal system to expands and the alveolar epithelium and the breast increases in size.^43^ When breastfeeding stops there is a regression in the breast tissue but there is no substantial reduction of the mammary glands.^44^ The lobules in the breast involute as a woman ages with reduction in number of alveoli. Over time there is a replacement of the mammary glands with fatty tissue.^44^

### 6.1 Gene‐expression studies

Studies have sought to understand the difference in gene‐expression in breast tissue from women who have had children compared to those that have not.^45^, ^46^, ^47^, ^48^ Two studies examined normal‐breast tissue from healthy‐postmenopausal volunteers using breast‐core‐needle biopsies.^45^, ^46^ These studies both found 208 genes to be differentially expressed and 96 genes overlapped between the studies. Genes that were altered were primarily related to RNA processing and cellular differentiation.^45^, ^46^ A study which used microdissection to isolate normal‐breast tissue from breast‐cancer patients also found similar changes in gene expression to the core‐needle‐biopsy studies.^47^ In contrast, a study which examined reduction‐mammoplasty samples did not find similar genes to the other studies.^48^ This may be due to differences in breast tissue in women who have larger breasts or that this study included both pre and post‐menopausal women.^48^ Only one gene was found across three or more of the gene‐expression studies, increased expression of TRAF3IP3.^45^, ^46^, ^48^ TRAF3‐interacting protein 3 is involved in cell maturation, tissue development, and immune response.^49^ Unfortunately, increased expression of TRAF3IP3 has been found in melanoma tumour samples and in the blood vessels of breast cancers.^49^, ^50^ Further research is needed to see if TRAF3IP3 plays a protective role in the parous breast.

### 6.2 Mammary‐epithelial stem cells

Terminal ductal lobuloalveolar units are regarded as the site of origin for the majority of human breast cancers and they contain mammary stem and progenitor cells.^51^ Mammary‐stem cells are typically involved in the homeostasis of the organ but also in promoting the elongation and development of the mammary ducts and alveoli during pregnancy.^52^ Studies in mice have found that an early pregnancy causes a persistent decrease in the number of functional mammary‐epithelial stem cells.^53^ The maintenance or differentiation of stem cells and their progenitors relies on regulation through gene expression, including chromatin modification, transcription factors, microRNAs and regulation through alternative RNA splicing.^54^, ^55^ The gene‐expression studies in the parous breast found genes primarily related to RNA processing and cellular differentiation.^45^, ^46^ Suggesting that the reduction in breast‐cancer risk associated with pregnancy may be associated with an alteration in maintenance or differentiation of breast cancer stem cells.

## 7 SUBTYPES OF BREAST CANCER

Breast cancer is categorised into three major subtypes based on the presence or absence of oestrogen receptor or progesterone receptor known as hormone receptors (HR) and human epidermal growth factor 2 (HER2).^56^ These three receptors are all growth factors, when overexpressed these stimulate the growth of cancer cells.^57^, ^58^, ^59^

### 7.1 HR+

Hormone‐receptor positive and HER2 negative is the most common type of breast cancer representing 70% of cases.^56^ When detected early and treated with endocrine therapy to block the hormone receptors,^60^ 99% of women survive 5 or more years.^56^ If the cancer is metastatic, that is spread beyond the breast, survival is typically 4–5 years.^56^

### 7.2 HER2+

HER2 positive breast cancers account for 15–20% of cases, about half of these cases are also hormone‐receptor positive. When detected early and treated with a combination of chemotherapy and HER2 inhibitors as well as endocrine therapy for those who are hormone‐receptor positive; 94% of women survive 5 or more years.^56^ If the cancer is metastatic survival is typically 4–5 years.^56^

### 7.3 Triple negative

Breast cancers that do not have hormone receptors or HER2. The cause of these cancers can be unknown but tumours from BRCA mutation carriers are typically within this subtype.^61^ This subtype has the poorest prognosis, when detected early and treated with chemotherapy only 85% of women survive 5 or more years. If the cancer is metastatic, survival is typically only 10–13 months.^56^

## 8 PREGNANCY AND SUBTYPES OF BREAST CANCER

### 8.1 HR+

Young age of first full‐term pregnancy (<25) was found to reduce the risk of HR+ breast cancer by 18–40% compared to women without children.^29^, ^62^ One or more full‐term pregnancy was found to reduce the risk of HR+ breast cancer by 29–35% compared to women without children.^63^, ^64^

### 8.2 HER2+

The literature on the risk of HER2+ breast cancer and pregnancy does not have a clear consensus. Some studies show an increased risk with one or more birth,^65^, ^66^ some no change^64^ and others a decreased risk.^63^

### 8.3 Triple negative

One or more full‐term pregnancy was found to reduce the risk of triple‐negative breast cancer by 30% in women aged 20–44 compared to women without children.^64^

### 8.4 BRCA1/2 carriers

Pregnancy is associated with a reduced risk of breast cancer in BRCA1/2 carriers, the greater number of pregnancies the larger the reduction in risk was observed.^34^, ^67^ A meta‐analysis has shown that a BRCA1/2 carrier needs to have three or more live births to reduce her breast‐cancer risk.^68^ Very‐young age at first full‐term pregnancy (<21 years) was found to decrease the risk of breast cancer by 9% for women with a BRCA1 mutation and by 17% for women with a BRCA2 mutation.^69^

## 9 BREASTFEEDING AND SUBTYPES OF BREAST CANCER

### 9.1 HR+

A 2015 meta‐analysis found no reduction in the risk for hormone‐receptor positive breast cancer associated with breastfeeding.^11^ There are a lot of studies on the topic and those that find a reduction in risk tend to be case–control studies where women with cancer are compared to age‐matched controls.^63^ In contrast, studies which follow a cohort of women over time in have not found a reduction in risk.^65^

### 9.2 HER2+

The studies that found an increased risk of HER2+ breast cancer with pregnancy, had a lower risk in women who breastfed.^65^, ^66^ The studies that found either no change or a decrease in risk associated with pregnancy also found no change with breastfeeding.^63^, ^64^

### 9.3 Triple negative

A 2015 meta‐analysis found a 20% reduction in the risk for triple‐negative breast cancer associated with breastfeeding.^11^ This reduction in risk was seen in both case–control and cohort studies.^11^ Another more recent study found a 191% increase in the risk of triple‐negative breast cancer in women who had children but did not breastfeed.^63^

### 9.4 BRCA mutations

Women with BRCA1 mutations who breastfed for more than one year were found to have a 22–50% reduced risk of breast cancer than those who never breastfed.^68^, ^70^ This large reduction in risk is notable as comes in a population that is at especially high risk of breast cancer. Women with BRCA1 mutations have a 65% risk of developing breast cancer by age 70.^71^ In contrast, there is no decreased risk associated with breastfeeding for women with BRCA2 mutations.^70^

Women with a family history of breast cancer should particularly be supported to breastfeed as a way of reducing their cancer risk. Many women are unaware of having a BRCA mutation and their increased cancer risk.^72^ Women with a carrier probability of less than 10% are currently ineligible to access free genetic testing for BRCA in the UK.^73^, ^74^ This is however, likely to change in the near future as the cost of genetic testing comes down.^72^

## 10 WHY DO WOMEN IN THE UK CHOOSE NOT TO BREASTFEED OR TO STOP BREASTFEEDING?

Breastfeeding is a highly‐emotive subject because so many women have not breastfed, or have experienced the pain of trying very hard to breastfeed and not succeeding.^75^ There are many reasons why women in high‐income countries choose not to breastfeed or to stop breastfeeding sooner than they intended to do so.^76^, ^77^

### 10.1 Insufficient milk

Public Health England recently studied mothers perceived barriers to breastfeeding.^78^ Mothers primary concern was worrying that the baby was getting enough milk or the right nutrients.^78^ Perceived insufficient milk supply is one of the top reasons that women stop breastfeeding^79^ although true‐milk insufficiency effects only around 10% of mothers.^80^ Many of the problems women experience with low‐milk supply has to do with how often milk is removed from the breast.^77^ If an infant is fed frequently and responsively this leads to a greater milk supply.^77^

### 10.2 Nipple pain and difficulties with latch

Problems can be caused by common conditions such as tongue‐tie in the infant^81^ and inverted nipples in the mother.^80^ Pain while breastfeeding can lead to women weaning their baby prematurely often in the first week after birth.^82^, ^83^

### 10.3 Professional support and public health policy

First‐time mothers, have little experience of seeing breastfeeding in their community and need additional support in positioning and attachment of the baby.^77^ Women benefit from the support of both professional and peer‐support breastfeeding networks^76^ and those who experience problems but did not get professional help were more likely to stop breastfeeding.^79^ This has been challenging in recent years due to the COVID‐19 pandemic.^84^

The “Ten Steps to Healthy Breastfeeding” are the basis of the WHO/UNICEF Baby‐Friendly Hospital Initiative^85^ (Box 1). However, there is little consensus on the most effective format to deliver prenatal breastfeeding education.^86^ Sweden has had 97% of its hospitals designated as Baby‐Friendly since 2000.^87^ In contrast, Ireland and the USA only have 47% and 12% of hospitals designated as Baby‐Friendly.^87^ The UK only has 43% of maternity services and 67% of health‐visiting services with full Baby‐Friendly accreditation.^88^ However, 95% of maternity services and 91% of health‐visiting services in the UK are working towards Baby‐Friendly accreditation.^88^

BOX 1 **Ten Steps to Successful Breastfeeding**

Every facility providing maternity services and care for new‐born infants should:

1. Have a written breastfeeding policy that is routinely communicated to all health care staff.

2. Train all health care staff in skills necessary to implement this policy.

3. Inform all pregnant women about the benefits and management of breastfeeding.

4. Help mothers initiate breastfeeding within a half‐hour of birth.

5. Show mothers how to breastfeed, and how to maintain lactation even if they should be separated from their infants.

6. Give new‐born infants no food or drink other than breastmilk, unless medically indicated.

7. Practice rooming‐in ‐ allow mothers and infants to remain together −24 hours a day.

8. Encourage breastfeeding on demand.

9. Give no artificial teats or pacifiers (also called dummies or soothers) to breastfeeding infants.

10. Foster the establishment of breastfeeding support groups and refer mothers to them on discharge from the hospital or clinic.

### 10.4 Family and friends attitudes to breastfeeding

Women do not breastfeed in isolation and rely on the advice and support of their family. A woman's partner is the most helpful or influential on feeding practices.^84^, ^89^ Mothers in the UK are more likely to breastfeed if they themselves were breastfed and most of their friends breastfeed.^79^ The advice women receive from their mother and mother‐in‐law is also very important.^77^, ^90^ However, this advice can often reflect cultural beliefs that aren't necessarily supportive of breastfeeding and may include outdated guidelines.^90^ The breastfeeding rates in the UK were even lower 30–40 years ago, 51% of women initiated breastfeeding in 1975 and 62% in 1990.^79^ As such the UK has a generation of grandmothers with less experience with successful breastfeeding than their daughters.

Sweden has historically high rates of breastfeeding initiation, which was 97% in 1990.^87^ As such Sweden is operating from a position of strength, the population has high levels of experience with breastfeeding. Conversely, The USA and Ireland had low rates of breastfeeding initiation in the 1990s (57% and 31.7%)^87^ and like the UK have had to build up experience in the population.

### 10.5 Public attitudes and legislation

Despite breastfeeding being protected by UK law^91^ women in the UK reported embarrassment over breastfeeding in public.^79^ Similar experiences have been reported in other high‐income countries such as Australia.^92^

### 10.6 Breastfeeding and the workplace

The longer the duration of a mother's maternity leave the longer the duration of breastfeeding.^93^ This is also associated with socioeconomic status, as women in higher‐income jobs may have additional maternity leave beyond the minimum statutory requirement or be more able to afford to take unpaid leave.

Breastfeeding in the workplace is not adequately protected in UK law. The law requires an employer to provide a space to rest including the ability to lie down.^94^ However, the law does not require the employer to grant paid breaks from work to express milk or breastfeed or provide facilities for the storage of expressed milk.^94^ This lack of legislation in the UK is a barrier to breastfeeding particularly for women who work in complex environments such as the armed forces.^95^ In contrast, the legislation in 121 countries provide for paid or unpaid breaks for lactating mothers, including the United States and Ireland.^96^ Using a lactation room in the workplace has been associated with longer duration of breastfeeding in working mothers.^97^

### 10.7 Promotion of formula milk

Formula milk is an essential product for children with complex medical needs and for those women who cannot breastfeed.^98^ However, decisions on the use of infant formula should be based on evidence and not advertising.^98^ The World Health Organisation launched an International Code of Marketing Breastmilk substitutes in 1981, it explicitly bans advertising and promotion to the general public.^99^ 70% of countries have adopted legal measures to implement the code.^100^ It is illegal in the UK to promote formula‐milk aimed at infants less than 6 months old, but the advertising of follow‐on milks for infants over 6 months is permitted.^101^ The UK is similar to most of Europe in that it has very few parts of the WHO Code adopted in law.^100^ Countries with similar laws to the UK that were enacted at a similar time have very divergent breastfeeding rates Sweden (98%) vs Ireland (55%).^20^, ^100^ Countries with no laws around the advertising of formula also have very divergent rates of breastfeeding Australia (92%) vs USA (79%).^20^, ^100^ Clearly, laws about the advertising of formula cannot explain the diversity of breastfeeding rates in high‐income countries. However, countries where the rates of breastfeeding are lower like the UK and Ireland are more likely to benefit from stricter legislation.

## 11 CONCLUSION

Breastfeeding reduces the risk of breast cancer by 4.3% for every 12 months of breastfeeding, which is in addition to the 7.0% decrease in risk observed for each birth. Breastfeeding has been shown to primarily reduce the risk of Triple‐Negative Breast Cancer (20%) as well as in carriers of BRCA1 mutations (22–50%). Women with a family history of breast cancer should particularly be supported to breastfeed as a way of reducing their cancer risk.

The molecular mechanisms of reduced breast‐cancer risk as a result of pregnancy appear to be related to RNA processing and cellular differentiation and may be associated with an alteration in maintenance or differentiation of breast cancer stem cells.

The UK has a low rate of breastfeeding in part due to a lack of experience in the population. An estimated 4.7% of breast cancer cases in the UK are caused by not breastfeeding. Expansion of the Baby‐Friendly Hospital Initiative, stricter laws on the advertising of formula milk and legislation to support nursing mothers in the workplace have the potential to increase breastfeeding rates in the UK.

## AUTHOR CONTRIBUTIONS

**Britta Stordal:** Funding acquisition (lead); investigation (lead); methodology (lead); writing – original draft (lead); writing – review and editing (lead).

## CONFLICT OF INTEREST

The author has no financial or non‐financial interests related to the work submitted for publication. The author states that there is no conflict of interest.

## ETHICS APPROVAL

This study was approved by the Natural Science Research Ethics Committee at Middlesex University. This study did not involve human participants or patient material, so no written informed consent was required.

## Supporting information


Appendix S1

## Data Availability

N/A
