# Supplementary material for: Breastfeeding reduces the risk of breast cancer: A call for action in high‐income countries with low rates of breastfeeding
Source: Cancer Med. 2022 Sep 26;12(4):4616–25. doi: 10.1002/cam4.5288 (PMC9972148; doi:10.1002/cam4.5288)
Supplement: Supplementary file 1 — Appendix S1 [file CAM4-12-4616-s001.docx]

**Supplementary Information - Methods**

**Relative Risk Reduction or Increase -** Publications reporting on the risk of breast cancer or other diseases typically report using risk ratios (RR), odds ratios (OR) or hazard ratios (HR). This research was commissioned by the charity Breast Cancer UK and is intended to be read by an audience with a secondary school knowledge of biology. We have taken a consistent and simplified approach to the way risk is reported in this review and converted the all the data from the literature pertaining to breast cancer risk into a relative risk reduction (RRR) or increase. Relative risk reduction can be calculated from risk ratios using Formula 1 below.

**Formula 1** RRR = 100% (1-RR)

**Odds Ratio** – The estimated lifetime risk of being diagnosed with breast cancer is 1 in 7 (15%) for women in the UK born after 1960. ^1^ Odds ratios approximate risk ratios when the outcome under consideration is rare, but can differ substantially when the outcome is common.^2^ Breast cancer occurs too frequently for odds ratios to be interpreted as if they were a risk ratio. Odds ratios were converted to risk ratios using the formula 2 ^3^ below and the specific population breast cancer risk described in Table 1. If a population did not have either an easily calculated breast cancer risk or one easily identified from the literature we did not convert the odds ratio. Risk ratios were then converted to a relative risk reduction or increase using the method above.

**Formula 2** - RR = OR(1 -P_0_) + (P_0_ x OR)

**Table 1 - Breast Cancer Risk in Various populations for the Conversion of Odds Ratios**

| **Population** | **Breast Cancer Risk** | **References Converted** |
| --- | --- | --- |
| Women (All Ages) | 13.6% lifetime cumulative risk | ^4,5^ |
| Women 55-64 Years | 5.5% Average cumulative lifetime risk of this age group. | ^6^ |
| Women 20-44 Years | 0.4% Average cumulative lifetime risk of this age group. | ^7^ |
| BRCA1 Mutation Carriers | 65% ^8^  Cumulative lifetime risk to age 70 | ^9,10^ |
| BRCA2 Mutation Carriers | 45% ^8^  Cumulative lifetime risk to age 70 | ^10^ |

Lifetime cumulative risk and average cumulative lifetime risk of various age groups was calculated using UK female breast cancer incidence ^11^ and mortality data ^12^ 2016-2018 with UK female population ^13^ and death data^14^ using Cancer Research UK’s lifetime risk calculator ^15^ adjusting for multiple primaries.^16^

**Hazard Ratio** – It is common in clinical papers to convert a hazard ratio of 0.6 for example, to a 40% reduction in the risk of death using the formula (100 x (1-HR) %). ^17^ However, this formula always leads to an overinterpretation of the change in risk ^17^ as hazard ratios are highly dependent on the length of follow up in the given study. Where studies have used hazard ratios we have reported their results as the percentage of subjects at a given time point who are cancer free.^18^ Some studies using hazard ratios have not provided enough information to report their results in this manner. ^19,20^ In this case we have reported their findings when significant as increased or decreased risk and not commented on the magnitude of the change. ^21^

**References**

1. Smittenaar CR, Petersen KA, Stewart K, et al. Cancer incidence and mortality projections in the UK until 2035. *Br J Cancer* 2016; 115: 1147–1155.

2. VanderWeele TJ. Optimal approximate conversions of odds ratios and hazard ratios to risk ratios. *Biometrics* 2020; 76: 746–752.

3. Zhang J, Yu KF. What’s the relative risk? A method of correcting the odds ratio in cohort studies of common outcomes. *JAMA* 1998; 280: 1690–1691.

4. Islami F, Liu Y, Jemal A, et al. Breastfeeding and breast cancer risk by receptor status--a systematic review and meta-analysis. *Ann Oncol* 2015; 26: 2398–2407.

5. Holm J, Eriksson L, Ploner A, et al. Assessment of Breast Cancer Risk Factors Reveals Subtype Heterogeneity. *Cancer Res* 2017; 77: 3708–3717.

6. Lord SJ, Bernstein L, Johnson KA, et al. Breast cancer risk and hormone receptor status in older women by parity, age of first birth, and breastfeeding: a case-control study. *Cancer Epidemiol Biomarkers Prev* 2008; 17: 1723–1730.

7. Li CI, Beaber EF, Tang M-TC, et al. Reproductive factors and risk of estrogen receptor positive, triple-negative, and HER2-neu overexpressing breast cancer among women 20-44 years of age. *Breast Cancer Res Treat* 2013; 137: 579–587.

8. Antoniou A, Pharoah PDP, Narod S, et al. Average risks of breast and ovarian cancer associated with BRCA1 or BRCA2 mutations detected in case Series unselected for family history: a combined analysis of 22 studies. *Am J Hum Genet* 2003; 72: 1117–1130.

9. Jernström H, Lubinski J, Lynch HT, et al. Breast-feeding and the risk of breast cancer in BRCA1 and BRCA2 mutation carriers. *J Natl Cancer Inst* 2004; 96: 1094–1098.

10. Evans D, Harkness E, Howel S, et al. Young age at first pregnancy does protect against early onset breast cancer in BRCA1 and BRCA2 mutation carriers. *Breast Cancer Res Treat* 2018; 167: 779–785.

11. *Breast Cancer Incidence by Age*. Cancer Research UK, https://www.cancerresearchuk.org/health-professional/cancer-statistics/statistics-by-cancer-type/breast-cancer/incidence-invasive#heading-One (2019, accessed 4 April 2022).

12. *Breast Cancer Mortality by Age*. Cancer Research UK, https://www.cancerresearchuk.org/health-professional/cancer-statistics/statistics-by-cancer-type/breast-cancer/mortality#heading-One (2019, accessed 4 April 2022).

13. *Estimates of the population for the UK, England and Wales, Scotland and Northern Ireland - Mid 2017*. Office for National Statistics, https://www.ons.gov.uk/peoplepopulationandcommunity/populationandmigration/populationestimates/datasets/populationestimatesforukenglandandwalesscotlandandnorthernireland (2017, accessed 4 April 2022).

14. *Deaths registered by single year of age, UK*. Office for National Statistics, https://www.ons.gov.uk/peoplepopulationandcommunity/birthsdeathsandmarriages/deaths/datasets/deathregistrationssummarytablesenglandandwalesdeathsbysingleyearofagetables (2017, accessed 4 April 2022).

15. *Lifetime Risk Calculator*. Cancer Research UK, https://www.cancerresearchuk.org/health-professional/cancer-statistics/cancer-stats-explained/our-calculations-explained#heading-Eight (2022, accessed 4 April 2022).

16. Sasieni PD, Shelton J, Ormiston-Smith N, et al. What is the lifetime risk of developing cancer?: the effect of adjusting for multiple primaries. *Br J Cancer* 2011; 105: 460–465.

17. Sashegyi A, Ferry D. On the Interpretation of the Hazard Ratio and Communication of Survival Benefit. *Oncologist* 2017; 22: 484–486.

18. Hernán MA. The Hazards of Hazard Ratios. *Epidemiology* 2010; 21: 13–15.

19. Nichols HB, Schoemaker MJ, Cai J, et al. Breast Cancer Risk After Recent Childbirth: A Pooled Analysis of 15 Prospective Studies. *Ann Intern Med* 2019; 170: 22–30.

20. Toss A, Grandi G, Cagnacci A, et al. The impact of reproductive life on breast cancer risk in women with family history or BRCA mutation. *Oncotarget* 2017; 8: 9144–9154.

21. Jordan SJ, Wilson LF, Nagle CM, et al. Cancers in Australia in 2010 attributable to total breastfeeding durations of 12 months or less by parous women. *Australian and New Zealand Journal of Public Health* 2015; 39: 418–421.
